# Treatment Options for Patients With Moyamoya Disease: A Retrospective Cohort Study

**DOI:** 10.31083/RN45032

**Published:** 2025-12-25

**Authors:** Yuting Luo, Chutong Guo, Shaoqing Wu, Xunsha Sun

**Affiliations:** ^1^Department of Neurology, The First Affiliated Hospital, Sun Yat-sen University, 510080 Guangzhou, Guangdong, China; ^2^Guangdong Provincial Key Laboratory of Diagnosis and Treatment of Major Neurological Diseases, 510080 Guangzhou, Guangdong, China; ^3^National Key Clinical Department and Key Discipline of Neurology, 510080 Guangzhou, Guangdong, China; ^4^Guangzhou Women and Children's Medical Center, Guangzhou Medical University, 510623 Guangzhou, Guangdong, China

**Keywords:** Moyamoya disease, human, surgery, Enfermedad de Moyamoya, humano, cirugía

## Abstract

**Purpose:**

Surgical revascularization is the preferred treatment for most patients with Moyamoya disease (MMD). Nevertheless, a considerable number of eligible patients choose non-surgical management. This study aimed to identify factors influencing treatment decisions, with particular emphasis on asymptomatic MMD patients.

**Materials and Methods:**

We conducted a retrospective analysis of MMD patients without surgical contraindications treated at our center between 2010 and 2022. Baseline characteristics were compared using Wilcoxon rank-sum and chi-squared tests. Multivariable logistic regression was used to identify factors associated with treatment selection.

**Results:**

Among the 147 included patients, 62.6% underwent surgical treatment. Younger age (OR = 0.88, 95% CI: 0.88–0.94, *p* < 0.001), married status (OR = 653.3, 95% CI: 41.61–10,264.22, *p* < 0.001), and absence of hyperlipidemia (OR = 0.16, 95% CI: 0.03–0.85, *p* < 0.05) were significantly associated with choosing surgery. Asymptomatic patients underwent surgery at a higher rate than symptomatic patients (67.9% vs. 59.6%). Younger age was a significant predictor of surgical preference in both symptomatic and asymptomatic subgroups.

**Conclusion:**

Younger age is strongly associated with the choice of surgical treatment in MMD, including in asymptomatic cases.

## Abstract (es)

**Objetivo:**

La revascularización quirúrgica es el tratamiento más adecuado para la mayoría de los pacientes con enfermedad de Moyamoya (MMD, Moyamoya disease). Sin embargo, un número considerable de pacientes elegibles optan por un tratamiento no quirúrgico. El objetivo de este estudio fue identificar los factores que influyen en las decisiones terapéuticas, con especial énfasis en los pacientes con MMD asintomática.

**Materiales y Métodos:**

Llevamos a cabo un análisis retrospectivo de los pacientes con MMD sin contraindicaciones quirúrgicas tratados en nuestro centro entre los años 2010 y 2022. Las características iniciales se compararon mediante la prueba de suma de rangos de Wilcoxon y la prueba de la χ^2^. Se utilizó una regresión logística multivariable para identificar los factores asociados con la selección del tratamiento.

**Resultados:**

De los 147 pacientes incluidos, el 62,6 % se sometió a tratamiento quirúrgico. La edad menor (OR = 0,88, IC del 95 %: 0,88–0,94, *p* < 0,001), el estado civil casado (OR = 653,3, IC del 95 %: 41,61–10.264,22, *p* < 0,001) y la ausencia de hiperlipidemia (OR = 0,16, IC del 95 %: 0,03–0,85, *p* < 0,05) se asociaron claramente con la elección de la cirugía. Los pacientes asintomáticos se sometieron a cirugía en una proporción mayor que los pacientes sintomáticos (el 67,9 % frente al 59,6 %). La edad menor fue un predictor significativo de la preferencia quirúrgica tanto en el subgrupo sintomático como en el asintomático.

**Conclusión:**

La menor edad está fuertemente asociada con la elección del tratamiento quirúrgico en la MMD, incluso en los casos asintomáticos.

## 1. Introduction

Moyamoya disease (MMD) is a rare cerebrovascular disorder characterized by progressive stenosis of the terminal bilateral internal carotid arteries and proximal anterior and middle cerebral arteries accompanied by vascular network formation at the skull base [1]. In urban China, the estimated national crude incidence was 0.59 (95% CI: 0.49–0.68) per 100,000 person-years, and the prevalence was 1.01 (95% CI: 0.81–1.21) per 100,000 people in 2016 [2]. MMD represents a significant risk factor for stroke in pediatric and young adult populations. It is an important risk factor for stroke in children and young adults [3, 4]. Although its etiology and pathogenesis remain incompletely understood, surgical revascularization is currently the most established and effective intervention [5, 6]. Surgery has been shown to restore cerebrovascular reserve capacity in pediatric MMD patients, and reduce rebleeding rates and mortality in adults presenting with hemorrhagic onset [7, 8, 9]. Nevertheless, surgical treatment carries risks, including perioperative ischemia, cerebral hyperperfusion syndrome, and rupture of fragile collateral vessels [10, 11]. Non-surgical management—typically involving antiplatelet therapy, regular clinical and imaging surveillance, or watchful waiting. A considerable number of patients eligible for surgery initially opt for non-operative management [12].

Cerebrovascular diseases should be managed according to subtype-specific strategies [13]. MMD also can be classified as symptomatic or asymptomatic based on clinical presentation. Acute ischemic stroke in younger populations differs from that in older adults in the distribution of risk factors, stroke subtypes, stroke severity, etiology, and outcome [14]. Asymptomatic MMD patients exhibit no history of stroke or current neurological symptoms [15]. Their management diverges significantly from that of symptomatic patients [5, 16]. While aggressive surgical revascularization is recommended for symptomatic MMD presenting with cerebral ischemia, cerebral hemorrhage, and/or decreased cerebral blood flow reserve [5]. Despite the absence of overt clinical manifestations in the early stages of asymptomatic MMD, asymptomatic individuals still demonstrate deficits in cognitive functions including memory, calculation, and visuospatial abilities [17, 18]. Asymptomatic MMD patients are at risk for cerebrovascular events as the disease progresses, with a reported annual stroke risk of 1.4% under conservative management [15, 19]. Surgical intervention in asymptomatic MMD is associated with longer progression-free survival compared with conservative treatment [20]. However, the operation method and timing of surgical treatment have not yet been determined [21].

Current research predominantly focuses on post-treatment outcomes rather than on the factors influencing initial treatment selection and influencing factors of MMD patients, especially asymptomatic MMD patients. To address this gap, we conducted a retrospective analysis of clinical data from patients diagnosed with MMD at our institution from January 2010 to February 2022. We summarized and compared the clinical characteristics of patients who opted for surgical treatment versus those who chose non-surgical treatment. This study compares the clinical profiles of patients who elected for surgical versus non-surgical management, aiming to identify factors associated with treatment choice and to support clinical decision-making of MMD.

## 2. Material and Methods

### 2.1 Population

We conducted a retrospective review of patients diagnosed with MMD who were treated in our center from January 1, 2010, to February 28, 2022. The inclusion criteria were as follows: (1) Patients diagnosed with MMD according to established clinical guidelines; (2) Patients met the surgical indications for MMD, which included: ① Symptoms indicative of cerebral ischemia related to the disease; ② Evidence of cerebral blood flow perfusion and velocity in the affected regions; ③ Presence of disease-related intracerebral hemorrhage without identifiable alternative causes [22, 23, 24]. Exclusion criteria: (1) Patients with incomplete imaging data or clinical data; (2) Patients with autoimmune disease, meningitis, intracranial tumors, down syndrome, down syndrome, head irradiation, and sickle cell disease who should be diagnosed as Moyamoya syndrome; (3) Exclusion of other surgical contraindications include concomitant tumors and poor general condition. In our center, discussions regarding surgical treatment options were conducted by physicians holding the title of associate professor or higher. Patients were classified into the non-surgical group if their medical records contained no documentation of a revascularization procedure in our center’s medical record system during the study period.

### 2.2 Investigation Follow-up

We retrospectively collected and compiled basic demographic and clinical information from the patients, including gender, age, current residence, nationality, marital status, initial symptoms, whether accompanied by cerebrovascular risk factors (diabetes, hypertension, hyperlipidemia, smoking history (smokers who have smoked continuously or cumulatively for 6 months or more in their lifetime), drinking history (alcohol use in the past year)), treatment methods.

### 2.3 Statistical Methods

Continuous variables were reported as median or interquartile range (IQR), and categorical variables were described as n (%). The Wilcoxon rank sum test was used for continuous variables, and the chi-square test was used for categorical variables. The glm function was used to construct a binary logistic model, and the variables with *p* < 0.1 were included in the multivariate logistic regression. *p* < 0.05 was considered statistically significant. All data analysis was done by R (4.2.1, R Foundation for Statistical Computing, Vienna, Austria).

## 3. Results

A total of 147 MMD patients without surgical contraindications were included in the final analysis. Of these, 92 patients (62.6%) underwent surgical treatment, and 55 (37.4%) received conservative management (Table 1). Comparative analyses demonstrated that the surgical group was significantly younger than the non-surgical group (*p* < 0.001), had a higher proportion of married individuals (*p* < 0.001), and exhibited lower rates of hyperlipidemia (*p* < 0.05) and alcohol consumption (*p* < 0.05).

**Table 1.** **The baseline of the survey**.

Characteristics		Non-surgical treatment	Surgical treatment	*p*
n		55	92	
Male, n (%)		22 (40.0%)	48 (52.2%)	0.15
Live in Guangdong province, n (%)		47 (85.5%)	68 (73.9%)	0.10
Married, n (%)		28 (50.9%)	91 (98.9%)	<0.001***
Age/year, median (IQR)		49 (42, 55.5)	39.5 (27.75, 47)	<0.001***
Initial mRS ≥3, n (%)		12 (21.8%)	32 (34.8%)	0.10
Diabetes mellitus, n (%)		4 (7.3%)	7 (7.6%)	1.00
Hypertension, n (%)		12 (21.8%)	21 (22.8%)	0.89
Hyperlipidemia, n (%)		10 (18.2%)	4 (4.3%)	0.01*
Smoking history, n (%)		11 (20.0%)	12 (13.0%)	0.26
Drinking history, n (%)		13 (23.6%)	9 (9.8%)	0.02*
Stroke history, n (%)		13 (23.6%)	22 (23.9%)	0.97
Symptom				0.32
	symptomatic MMD, n (%)	38 (69.1%)	56 (60.9%)	
	asymptomatic MMD, n (%)	17 (30.9%)	36 (39.1%)	
Intracranial aneurysm, n (%)		6 (10.9%)	13 (14.1%)	0.57
Suzuki ≥3, n (%)		41 (74.5%)	65 (70.7%)	0.61
PCA steno-occlusion, n (%)		9 (16.4%)	7 (7.6%)	0.10
Bilateral hemispheres involvement, n (%)		48 (87.3%)	74 (80.4%)	0.29
To learn the knowledge of MMD, n (%)		44 (80.0%)	83 (90.2%)	0.08

Patients who opted for surgical treatment were younger (*p* < 0.001), had a higher proportion of married individuals (*p* < 0.001), had a lower prevalence of hyperlipidemia (*p* < 0.05), and drinking history (*p* < 0.05). IQR, Inter Quartile Range; mRS, Modified Rankin Scale; PCA, posterior cerebral artery; MMD, Moyamoya disease; *, *p* < 0.05; ***, *p* < 0.001.

Multivariate logistic regression analysis identified age, marital status, and hyperlipidemia as factors significantly associated with treatment selection of patients with MMD (Table 2). Younger age was independently associated with a greater likelihood of undergoing surgical intervention (OR = 0.88, 95% CI: 0.88–0.94, *p* < 0.001). Married patients were significantly more likely to opt for surgery (OR = 653.3, 95% CI: 41.61–10,264.22, *p* < 0.001). Conversely, the absence of hyperlipidemia was also associated with a higher probability of selecting surgical treatment (OR = 0.16, 95% CI: 0.03–0.85, *p* < 0.05).

**Table 2.** **Univariate and multivariate logistic analysis of the factors affecting the choice of surgical treatment in MMD**.

Characteristics	OR (95% CI) Univariate analysis	*p*	OR (95% CI) Multivariate analysis	*p*
Age/year	0.94 (0.91–0.97)	<0.001***	0.88 (0.88–0.94)	<0.001***
Male	1.64 (0.83–3.22)	0.15		
Married	87.75 (11.42–674.40)	<0.001***	653.53 (41.61–10,264.22)	<0.001***
Live in non-Guangdong province	2.07 (0.86–5.01)	0.11		
Initial mRS ≥3	1.91 (0.89–4.13)	0.10	1.49 (0.44–5.04)	0.52
Diabetes mellitus	1.05 (0.29–3.76)	0.94		
Hypertension	1.06 (0.47–2.37)	0.89		
Hyperlipidemia	0.21 (0.06–0.69)	0.01*	0.16 (0.03–0.85)	0.03*
Smoking history	0.60 (0.25–1.47)	0.26		
Drinking history	0.35 (0.14–0.87)	0.03	0.30 (0.09–1.03)	0.06
Stroke history	1.02 (0.46–2.23)	0.97		
Intracranial aneurysm	1.34 (0.48–3.77)	0.57		
Suzuki ≥3	0.82 (0.39–1.75)	0.61		
PCA steno-occlusion	0.42 (0.15–1.20)	0.11		
Involved hemispheres	1.67 (0.65–4.29)	0.29		
Knowledge of MMD	0.43 (0.17–1.13)	0.09	0.30 (0.07–1.40)	0.13
Symptom	0.84 (0.42–1.66)	0.62		

Patients were more likely to choose surgical treatment if they were younger (OR = 0.94, 95% CI: 0.91–0.97, *p* < 0.001), married (OR = 653.53, 95% CI: 41.61–10,264.22, *p* < 0.001), and had no hyperlipidemia (OR = 0.21, 95% CI: 0.06–0.69, *p* < 0.01). *, *p* < 0.05; ***, *p* < 0.001. OR, odds ratio.

The rate of surgical treatment was higher among asymptomatic MMD patients (67.9%, 36/53) than among symptomatic patients (59.6%, 56/94). Factors associated with treatment selection were further analyzed separately in symptomatic and asymptomatic subgroups. Multivariate analysis revealed that younger age was consistently associated with an increased likelihood of choosing surgical treatment in both symptomatic and asymptomatic patients (**Supplementary Tables 1,2**).

## 4. Discussion

In this study, 62.6% of patients with (MMD underwent surgical treatment, whereas 37.4% received conservative management. Younger age, married status, and the absence of hyperlipidemia were significantly associated with the selection of surgical intervention. The proportion of asymptomatic patients who underwent surgery was higher than that of symptomatic patients (67.9% vs. 59.6%). Younger age was a significant factor influencing the treatment choice in both symptomatic and asymptomatic MMD.

An inverse correlation was observed between patient age and the likelihood of undergoing surgery. A national Korean study reported a surgical rate of 66.3% in patients aged 0–14, compared to 21.5% in those older than 15, which is consistent with our findings [25]. Previous studies indicate that surgical revascularization in pediatric MMD significantly and durably reduces stroke risk, suggesting that intervention should be performed promptly [26, 27]. In adults with ischemic or hemorrhagic MMD, revascularization is also more effective than conservative management. However, elderly patients (over 50 years) typically present with ischemic symptoms and more advanced Suzuki stages [28]. Conservative management is often recommended for asymptomatic or clinically stable elderly patients, whereas the optimal surgical approach for symptomatic elderly individuals remains under investigation. Therefore, the description of the clinical characteristics of patients grouped by age is helpful for a better understanding of the complete clinical manifestations of this disease [29].

In our cohort, a greater proportion of asymptomatic patients (67.9%) underwent surgery compared to symptomatic patients (59.6%). The therapeutic benefit of surgery for symptomatic MMD is well-established. By contrast, the benefits of revascularization for asymptomatic adults remain unclear. On one hand, asymptomatic MMD is a progressive disease, and studies have shown that patients remain at risk for cerebrovascular events [15, 30]. Conservative management alone may therefore be insufficient to halt disease progression. The primary goal of MMD treatment is to reduce the risk of complications, including cerebral infarction and hemorrhage. On the other hand, advances in diagnostic and surgical techniques are improving the safety and efficacy of MMD management. For instance, high-resolution magnetic resonance imaging (MRI) can effectively differentiate MMD from intracranial atherosclerotic disease [31]. Ultra-high-field MRI (e.g., 7 Tesla) may provide further clarification of intracranial vascular pathology [32]. Furthermore, progress in surgical techniques continues to mitigate procedure-related risks. Techniques such as “awake” craniotomy can significantly reduce the risk of intraoperative ischemia [33, 34, 35]. Continuous, real-time monitoring of cerebral autoregulation may help prevent postoperative complications [32]. Ongoing refinements in treatment concepts—including surgical timing, technique selection, and perioperative management—are contributing to more standardized and effective care [16]. Therefore, future management of MMD should emphasize innovation in diagnostic and surgical technologies, as well as the refinement of treatment strategies, particularly for asymptomatic disease.

No significant differences in conventional cerebrovascular risk factors, presenting symptoms, or imaging stages were observed between the surgical and non-surgical groups. Several factors may explain these findings. First, most symptomatic patients present at Suzuki stages III–IV, which limits more granular clinical stratification for treatment guidance [16]. Second, the pathophysiology of MMD (involving stenosis and neovascularization) is distinct, and traditional cerebrovascular risk factors may not be the primary drivers of MMD-specific stroke risk [21].

This study aimed to identify factors associated with treatment selection in MMD; however, several limitations must be acknowledged. First, this single-center study has a limited sample size and geographic scope, which may affect the generalizability of the findings. Future multi-center studies with larger samples are needed to validate the influence of marital status on decision-making. Second, technical aspects of care, including specific surgical procedures and perioperative nursing protocols, were not analyzed. Furthermore, our data cannot account for temporal trends in clinical practice—such as the adoption of advanced imaging, refined surgical techniques, and enhanced multidisciplinary expertise—that may have influenced treatment decisions and outcomes over the 12-year study period. Third, the retrospective design inherently carries a risk of incomplete data and unmeasured confounding. Details of clinician-patient communication were unavailable, precluding assessment of adherence to guidelines or the potential influence of physician bias on treatment recommendations. Fourth, due to data constraints, this analysis was limited to clinical baseline characteristics, offering only a partial perspective on treatment selection. Socioeconomic factors—such as insurance type, household income, and out-of-pocket costs—which may strongly influence decision-making, were not available. Future prospective studies should integrate clinical, socioeconomic, and cultural data within multi-center frameworks to better understand treatment selection in MMD.

## 5. Conclusion

The younger the patients with MMD, the more likely they are to choose surgery, even for asymptomatic MMD.

## Data Availability

The corresponding author can be contacted for further information regarding the data generated or analyzed during this study.

## Abbreviations

MMD, Moyamoya disease; CI, Confidence interval; OR, Odds ratio.

## Supplementary Material

Supplementary material associated with this article can be found, in the online version, at https://doi.org/10.31083/RN45032.
