# Supplementary material for: Treatment Options for Patients With Moyamoya Disease: A Retrospective Cohort Study
Source: Rev Neurol. 2025 Dec 25;80(12):45032. doi: 10.31083/RN45032 (PMC12781205; doi:10.31083/RN45032)
Supplement: Supplementary file 1 [file 1576-6578-80-12-45032-s1.docx]

Supplementary Table 1: Univariate and multivariate logistic analysis of the factors affecting the choice of surgical treatment in asymptomatic MMD

| Characteristics | OR(95% CI) Univariate analysis | *p* | OR(95% CI) Multivariate analysis | *p* |
| --- | --- | --- | --- | --- |
| Male | 3.20 (1.05 - 9.74) | 0.04* | 2.79 (0.80 - 9.66) | 0.11 |
| Age/year | 0.95 (0.91 - 0.99) | 0.01* | 0.95 (0.91 - 0.99) | 0.02* |
| Live in non-Guangdong province | 2.07 (0.50 - 8.54) | 0.32 |  |  |
| Initial mRS≥3 | 2.07 (0.50 - 8.54) | 0.32 |  |  |
| Diabetes mellitus | 8883204.77 (0.00 - Inf) | 0.99 |  |  |
| Hypertension | 2.07 (0.50 - 8.54) | 0.32 |  |  |
| Hyperlipidemia | 0.23 (0.04 - 1.38) | 0.11 |  |  |
| Smoking history | 1.09 (0.18 - 6.48) | 0.93 |  |  |
| Drinking history | 1.09 (0.18 - 6.48) | 0.93 |  |  |
| Stroke history | 0.35 (0.07 - 1.76) | 0.21 |  |  |
| Intracranial aneurysm | 1.09 (0.18 - 6.48) | 0.93 |  |  |
| suzuki≥3 | 0.63 (0.19 - 2.09) | 0.45 |  |  |
| PCA steno-occlusion | 0.08 (0.01 - 0.78) | 0.03 | 0.15 (0.02 - 1.60) | 0.12 |
| Lateral hemispheres involved | 1.73 (0.32 - 9.43) | 0.53 |  |  |

Age was an independent factor in the choice of surgical treatment in patients with MMD. mRS: Modified Rankin Scale; PCA: posterior cerebral artery; MMD: Moyamoya disease; *: *p*＜0.05.

Supplementary Table 2: Univariate and multivariate logistic analysis of the factors affecting the choice of surgical treatment in symptomatic MMD

| Characteristics | OR(95% CI) Univariate analysis | *p* | OR(95% CI) Multivariate analysis | *p* |
| --- | --- | --- | --- | --- |
| Male | 1.05 (0.44 - 2.49) | 0.92 |  |  |
| Age/year | 0.93 (0.88 - 0.97) | 0.002** | 0.89 (0.82 - 0.96) | 0.002** |
| Live in non-Guangdong province | 2.08 (0.67 - 6.44) | 0.20 |  |  |
| Married | 36.400 (4.49 - 295.25) | < 0.001*** | 257.34 (12.60 - 5257.25) | < 0.001*** |
| Initial mRS≥3 | 1.97 (0.77 - 5.03) | 0.16 |  |  |
| Diabetes mellitus | 0.78 (0.19 - 3.14) | 0.73 |  |  |
| Hypertension | 0.73 (0.27 - 2.00) | 0.54 |  |  |
| Hyperlipidemia | 0.18 (0.04 - 0.97) | 0.05* | 0.12 (0.02 - 1.02) | 0.05* |
| Smoking history | 0.49 (0.17 - 1.44) | 0.20 |  |  |
| Drinking history | 0.22 (0.07 - 0.70) | 0.01* | 0.24 (0.05 - 1.14) | 0.07 |
| Stroke history | 1.55 (0.60 - 4.00) | 0.36 |  |  |
| Intracranial aneurysm | 1.53 (0.43 - 5.44) | 0.51 |  |  |
| suzuki≥3 | 1.00 (0.38 - 2.66) | 1.00 |  |  |
| PCA steno-occlusion | 0.96 (0.25 - 3.68) | 0.95 |  |  |
| Lateral hemispheres involved | 1.70 (0.54 - 5.34) | 0.37 |  |  |

Age and marital status can affect the treatment decisions of MMD patients. Younger and married patients were more likely to choose surgical treatment. mRS: Modified Rankin Scale; PCA: posterior cerebral artery; *: *p* < 0.05; **: P < 0.001.
